# Rapid Developability Assessments to Formulate Recombinant Protein Antigens as Stable, Low-Cost, Multi-Dose Vaccine Candidates: Case-Study With Non-Replicating Rotavirus (NRRV) Vaccine Antigens

**DOI:** 10.1016/j.xphs.2020.11.039

**Published:** 2021-03

**Authors:** Nishant Sawant, Kawaljit Kaur, David A. Holland, John M. Hickey, Sanjeev Agarwal, Joseph R. Brady, Neil C. Dalvie, Mary Kate Tracey, M. Lourdes Velez-Suberbie, Stephen A. Morris, Shaleem I. Jacob, Daniel G. Bracewell, Tarit K. Mukhopadhyay, Kerry R. Love, J. Christopher Love, Sangeeta B. Joshi, David B. Volkin

**Affiliations:** a Department of Pharmaceutical Chemistry, Vaccine Analytics and Formulation Center, University of Kansas, 2030 Becker Drive, Lawrence, KS 66047, USA; b Department of Chemical Engineering, Koch Institute for Integrative Cancer Research, Massachusetts Institute of Technology, Cambridge, MA 02139, USA; c Department of Biochemical Engineering, University College London, Bernard Katz Building, Gower Street, London WC1E 6BT, UK

**Keywords:** Developability, Vaccine, Formulation, Stability, Multi-dose, Preservative, Rotavirus

## Abstract

A two-step developability assessment workflow is described to screen variants of recombinant protein antigens under various formulation conditions to rapidly identify stable, aluminum-adjuvanted, multi-dose vaccine candidates. For proof-of-concept, a series of sequence variants of the recombinant non-replicating rotavirus (NRRV) P[8] protein antigen (produced in *Komagataella phaffii*) were compared in terms of primary structure, post-translational modifications, antibody binding, conformational stability, relative solubility and preservative compatibility. Based on these results, promising P[8] variants were down-selected and the impact of key formulation conditions on storage stability was examined (e.g., presence or absence of the aluminum-adjuvant Alhydrogel and the preservative thimerosal) as measured by differential scanning calorimetry (DSC) and antibody binding assays. Good correlations between rapidly-generated developability screening data and storage stability profiles (12 weeks at various temperatures) were observed for aluminum-adsorbed P[8] antigens. These findings were extended and confirmed using variants of a second NRRV antigen, P[4]. These case-study results with P[8] and P[4] NRRV variants are discussed in terms of using this vaccine formulation developability workflow to better inform and optimize formulation design with a wide variety of recombinant protein antigens, with the long-term goal of rapidly and cost-efficiently identifying low-cost vaccine formulations for use in low and middle income countries.

## Introduction

Vaccination is a cornerstone of public health for preventing illness and death due to common infectious diseases. Despite ongoing improvements in global vaccination coverage, according to the World Health Organization (WHO), an estimated 19.4 million infants still did not receive routine immunizations in 2018 with ~60% of them residing in developing countries.^1^ One key approach to enhance access and improve global vaccination rates is to lower vaccine manufacturing costs, while at the same time, expanding production capacity to maintain a consistent supply.^2^ Concomitantly, there is a promising trend towards developing new vaccine candidates using recombinant subunit protein antigens designed by reversed vaccinology,^3^ which not only offers advantages of improved safety and efficacy, but also enhanced manufacturing scalability and lower costs compared to the traditional approaches (i.e., live, attenuated or inactivated versions of viral or bacterial pathogens).^4^ The major cost categories associated with manufacturing such recombinant protein antigens include production facilities and their maintenance, labor, equipment and quality control.^2^ Here, we sought to develop a framework to rapidly and cost-efficiently address challenges in developing low-cost formulations of recombinant protein vaccine candidates including using (1) adjuvants to augment immune responses resulting in antigen sparing (i.e., more doses per mg of antigen),^5^, ^6^, ^7^ and (2) preservatives to develop multi-dose presentations (i.e., more doses per vial of vaccine).

Our goal was to design a vaccine formulation developability assessment work-flow to rapidly screen different recombinant protein antigens and formulation conditions using minimal material to identify optimally stable, low-cost vaccine dosage forms targeted for use in low and middle income countries (LMICs). As a proof-of-concept, we focused on non-replicating rotavirus (NRRV) recombinant protein antigens. The preformulation characterization and formulation development of three different *E. coli* expressed recombinant NRRV protein antigens (P[8], P[6] and P[4]; see nomenclature below) has been recently described.^8^, ^9^, ^10^, ^11^ In this work, the NRRV antigens were a good case study to assess the utility of newer, more rapid formulation developability assessments (evaluating multiple antigen variants available only in small quantities) compared to more standard, time-consuming formulation development approaches (with preselected antigens available in larger quantities).

The NRRV subunit vaccine candidate was initially developed at the NIH^12^^,^^13^ and is currently in mid to late stage clinical trials as sponsored by PATH.^14^^,^^15^ The vaccine in clinical development comprises three subunit protein antigens produced and purified from *E. coli*. Each NRRV antigen is a recombinant fusion-protein consisting of a truncated version of the rotavirus surface protein VP4 (known as VP8), genetically fused to the tetanus toxoid universal CD4^+^ T-cell epitope (P2). The three fusion-protein antigens derive from the VP8 component of three different RV serotypes, P[8], P[6], and P[4]. This leads to the nomenclature P2–VP8–P[8], P2–VP8–P[6], and P2–VP8–P[4], which for simplicity, are often referred to as P[8], P[6], and P[4], respectively. The physicochemical properties of these three NRRV antigens are described in detail elsewhere.^9^

The development of the series of NRRV antigen variants from *K. phaffii*, in terms of design rationale, experimental strategies for low-cost vaccine production, and immunological characterization, are presented in detail elsewhere.^16^ We used these sequence variants of P[8] and P[4] NRRV as a case study to demonstrate proof-of-concept for a two-stage formulation developability assessment workflow to rapidly assess formulation variables for recombinant vaccine antigens that could be applied to accelerate development of low-cost vaccine dosage forms targeted for use in low and middle income countries (LMICs). Stage 1 evaluates the physicochemical and immunochemical binding properties of each NRRV protein antigen, and Stage 2 examines the impact of various storage temperatures on conformational stability and antibody binding of down-selected P[8] and P[4] NRRV variants, both in the presence and absence of an aluminum-adjuvant (Alhydrogel®, AH) and a vaccine preservative (thimerosal). Finally, to determine the predictability of developability data (Stage 1) to rank-order antigen storage stability as formulated multi-dose vaccine candidates (Stage 2), correlations were made between the developability datasets and the storage stability profiles of selected P[8] and P[4] NRRV variants in various aluminum adjuvanted, multi-dose formulations.

## Materials and Methods

### Materials

A total of five different NRRV P[8] fusion-protein antigens were used in these studies including *E*. *coli-*expressed parent protein P[8], *K. phaffii* (*P. pastoris*)-expressed parent protein P[8] with *N*-terminal truncations, *K. phaffii-*expressed parent protein P[8], *K. phaffii-*expressed P[8] double mutant (N85A, N151A), and *K. phaffii-*expressed P[8] triple mutant (N85Q, N151Q, C171S). These molecules are referred to as *E. coli* P[8], *Pp* P[8] (truncated), *Pp* P[8], *Pp* P[8]-N85A,N151A, and *Pp* P[8]-N85Q,N151Q,C171S, respectively. A total of three different NRRV P[4] fusion-protein antigens were used in these studies including *E*. *coli*-expressed parent protein P[4], *K. phaffii*-expressed parent protein P[4], and *K. phaffii-*expressed P[4] cysteine mutant (C173S), which are referred to as *E. coli* P[4], *Pp* P[4] and *Pp* P[4]-C173S, respectively. The design, preparation and characterization of these P[8] and P[4] variants expressed in *K. phaffii* are presented in detail elsewhere.^16^ Fed-batch production and purification of the NRRV P[8] and P[4] proteins are described in brief in the Supplemental Methods section and in detail elsewhere.^17^

Alhydrogel® adjuvant (2% aluminum hydroxide gel suspension, 10 mg/mL aluminum) was purchased from InvivoGen (San Diego, CA). Sodium chloride (NaCl) and sodium phosphate dibasic heptahydrate were purchased from Fisher Chemicals (Hampton, NH). Ammonium sulfate, 8-anilino-1-naphthalenesulfonic acid (ANS), thimerosal, dimethyl sulfoxide (DMSO), bovine serum albumin (BSA), and sodium phosphate monobasic monohydrate were purchased from Sigma-Aldrich (St. Louis, MO). Materials for SDS-PAGE, EZ-Link™ Sulfo–NHS–LC-biotinylation kit, casein blocking buffer, Tween 20, Slide-A-Lyzer mini dialysis devices, HPLC vials, LC-MS grade mobile phases, and isopropanol were obtained from Thermo Fisher Scientific (Waltham, MA). NRRV P[8] and P[4]- specific mAbs were developed by PATH and obtained from Precision Antibody (Columbia, MD) as described elsewhere.^8^^,^^11^

### Sample Preparation

The preparation of NRRV protein samples (with and without Alhydrogel® aluminum adjuvant) used for developability studies (Stage 1 testing) and storage stability studies (Stage 2 testing) is described in the Supplemental Methods Section. The various NRRV formulations contained 0.12 mg/mL NRRV protein (±1.125 mg/mL aluminum) in a 0.5 mM sodium phosphate, 150 mM NaCl buffer at pH 7.0 (±0.01% w/v thimerosal).

### Physicochemical Assays

Experimental details of the physicochemical methods used in this work have been described previously,^9^, ^10^, ^11^ and the experimental setups and analytical methods used are described in the Supplemental Methods section including UV–visible spectroscopy, SDS-PAGE, intact protein mass spectrometry, differential scanning calorimetry (DSC), extrinsic fluorescence spectroscopy, and the ammonium sulfate precipitation assay.

### Immunochemical Assays (Octet Biolayer Interferometry and ELISA)

Antibody binding of various P[8] molecules was determined using an Octet Red96 Biolayer Interferometry System (Pall Forte Bio LLC, Fremont, CA). The P[8]-specific mAb was biotinylated using EZ-Link™ Sulfo–NHS–LC-biotinylation kit following the manufacturer's instructions, aliquoted, and stored at 4 °C. Binding experiments were performed using high precision streptavidin biosensors (Forte Bio, Cat No. 18–5117) in 96-well black microplates (Greiner Bio-One). Assay kinetics buffer (1X PBS pH 7.2 + 0.5% BSA + 0.05% tween 20) was used for baseline, dissociation, and reference wells as well as for diluting the P[8]-specific antibody and various P[8] sample solutions. Biosensors were hydrated for ~15 min in kinetics buffer prior to the run. Binding assay for each sample was performed in triplicate using 1 μg/mL of P[8]-specific antibody and seven point 1:2 serial dilutions of P[8] with a starting concentration of 5 μg/mL. Association and dissociation steps were carried out at 1000 rpm for 300 and 600 s, respectively. Data analysis was performed using Octet Data Analysis software (v 10.0, Forte Bio). Following reference subtraction, baseline alignment, inter-step correction, and data processing with Savitzky-Golay filtering, association and dissociation traces of various P[8] samples were fit to a global 1:1 fitting model. Binding affinity and rate constants were extracted from the curve fitting analysis of the kinetics data. The inhibition ELISA assay used in this work, including the NRRV specific antibodies used and the nature of their interaction with NRRV antigens, is described in detail elsewhere (McAdams et al., manuscript in preparation) and briefly in the Supplemental Methods.

## Results

**Fig. 1:**
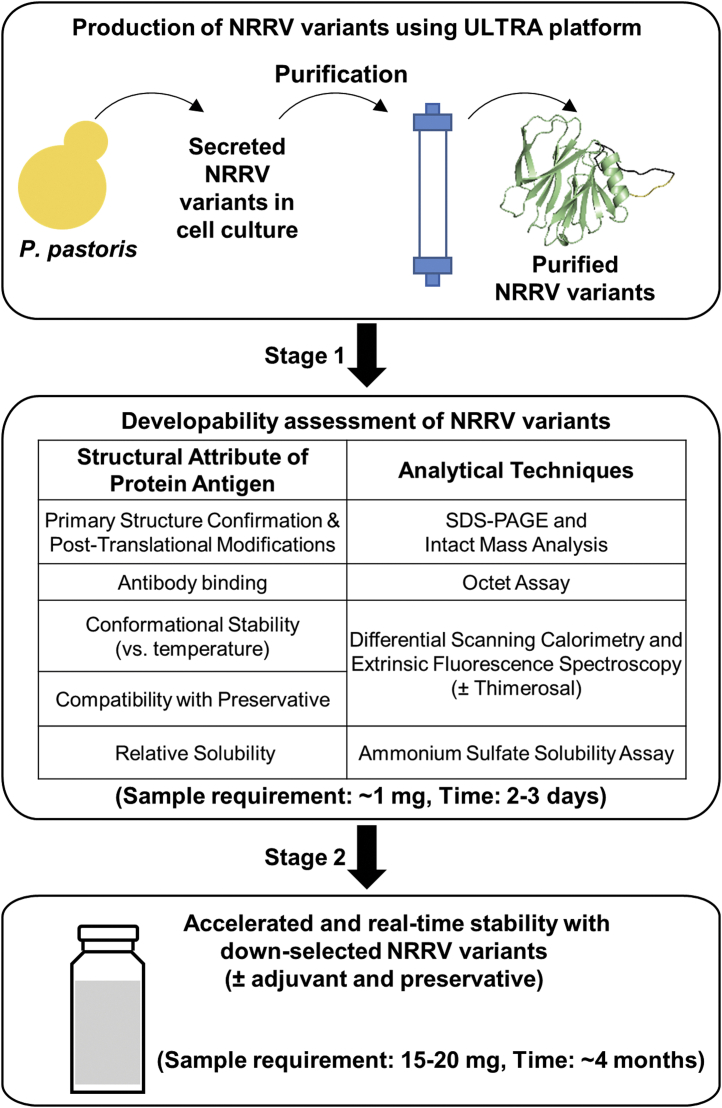
Developability assessment workflow for vaccine multi-dose formulation development as applied to evaluation of recombinant NRRV antigens (parent protein and sequence variants) produced in *K. phaffii*. Stage 1 physicochemical and immunochemical binding analyses are performed with antigens in solution, while Stage 2 stability studies are carried out with antigens in solution as well as bound to aluminum adjuvant.

We devised an overall two-step workflow for assessing the developability of recombinant protein antigens to be formulated as multi-dose vaccines, and used aluminum-adjuvanted subunit NRRV antigens as a model system (Fig. 1). The experimental outline begins with selection and production of each NRRV variant in *K. phaffii* followed by purification from the culture supernatant.^16^ High-throughput analytical techniques were then used to evaluate and compare various key structural attributes of the proteins including primary structure/post-translational modifications, antibody binding, conformational stability (±the preservative thimerosal), and relative solubility. Stage 1 data were generated in 2–3 days using only ~1 mg of each variant in solution (no aluminum adjuvant). Promising NRRV variants produced in *K. phaffii* were then down-selected for storage stability assessments requiring additional material (~15–20 mg). In Stage 2, accelerated and real-time 3-month stability studies were performed with and without preservative (thimerosal) and adjuvant (Alhydrogel®, AH). Although the combined Stage 1 and 2 developability assessments were completed in ~4 months, the timeline in this case study benefited from previous work in our lab on the physicochemical characterization and formulation development of three different *E. coli* expressed recombinant NRRV protein antigens (P[8], P[6] and P[4]),^9^, ^10^, ^11^ especially in terms of availability of immunological reagents required for antigen-antibody binding studies.

### Stage 1 Developability Assessments of NRRV P[8] Parent Protein and Variants

A total of five different NRRV P[8] antigens were evaluated, including the parent protein and variants produced in two expression systems *E. coli* and *K. phaffii* (*P. pastoris*, *Pp*). Samples included the parent protein *E. coli* P[8], and *Pp* P[8], a truncated version of the parent protein *Pp* P[8] (truncated), and two molecular engineered variants *Pp* P[8]-N85A,N151A and *Pp* P[8]-N85Q,N151Q,C171S. We characterized the five P[8] antigens as baseline information describing purity/potency, compatibility with preservatives, inherent conformational stability and relative solubility as the first step (Stage 1) of the developability workflow for multi-dose vaccine formulation development (Fig. 1).

**Fig. 2:**
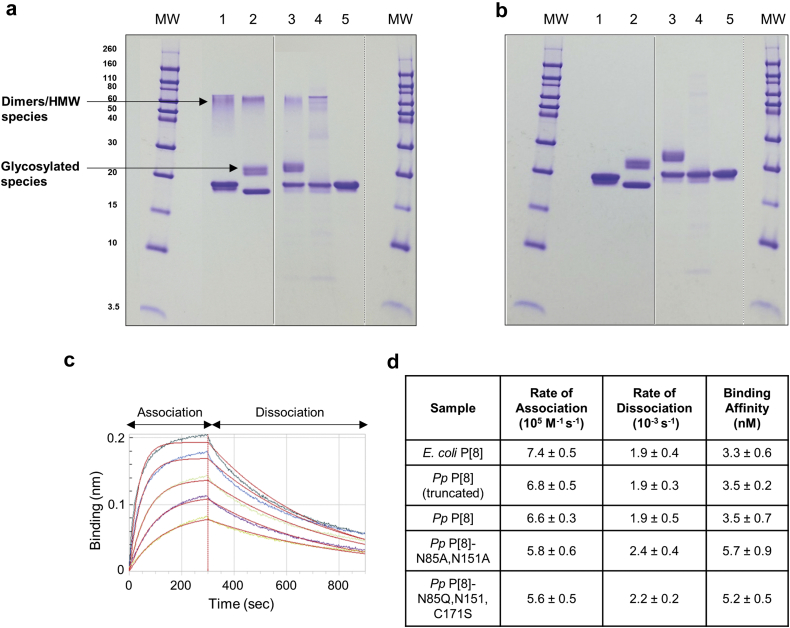
Characterization of five NRRV P[8] antigens evaluated in the vaccine formulation developability assessments. SDS-PAGE analysis of purity and composition of the NRRV P[8] parent antigen and variants under (a) non-reducing and (b) reducing conditions. Immunochemical binding analysis of P[8] antibody binding to NRRV P[8] parent antigen and variants as measured by BLI including (c) Representative sensorgram for *Pp* P[8]-mAb interaction, and (d) Binding and kinetic parameters measured using Octet Red96 instrument (values shown are n = 3, 1 SD). For gels, lanes include (MW) Molecular Weight marker in kDa, (1) *E. coli* P[8], (2) *Pp* P[8] (truncated), (3) *Pp* P[8], (4) *Pp* P[8]-N85A,N151A, and (5) *Pp* P[8]-N85Q,N151Q,C171S. Solid dividing line: images of two different gels; and dashed dividing line: images of different parts of same gel. The presence of *N*-linked glycosylated species was confirmed by Endo H enzyme treatment as shown in Supplementary Fig. S1.

The purity and composition (SDS-PAGE) as well as antibody binding activity (Bio-layer Interferometry, BLI) of the five P[8] antigens were determined (Fig. 2). The five P[8] samples showed high purity (~90–95% by SDS-PAGE), and had minimal levels of residual host cell protein and DNA (data not shown). A major band at ~20 kDa was observed for four of the P[8] proteins (*E. coli* P[8], *Pp* P[8], *Pp* P[8]-N85A,N151A, and *Pp* P[8]-N85Q,N151Q,C171S), while the band for *Pp* P[8] (truncated) migrated slightly lower than 20 kDa as expected (Fig. 2a and b). Bands corresponding to glycosylated species were observed between 20 and 30 kDa for both truncated and full-length *Pp* P[8] samples (*N*-linked glycosylation was confirmed by Endo-H enzyme treatment, Supplementary Fig. S1). As expected, these bands were absent in the variants (*Pp* P[8]-N85A,N151A and *Pp* P[8]-N85Q,N151Q,C171S) lacking the *N*-linked Asn glycosylation sites. Finally, additional higher molecular bands (HMW) corresponding to disulfide linked dimers/oligomeric species were observed between 40 and 80 kDa for four of the five P[8] protein antigens, except for variant without the single cysteine residue *Pp* P[8]-N85Q,N151Q,C171S, under non-reducing conditions and were absent under reducing conditions (Fig. 2a vs 2b). The overall structural integrity and antibody binding of the five P[8] antigens was evaluated using BLI. Fig. 2c shows representative association and dissociation binding curves for *Pp* P[8] with a P[8]-specific antibody. Overall, the five P[8] protein antigens displayed similar *in vitro* antigenicity in terms of rates of association and dissociation as well as corresponding binding affinity as displayed in Fig. 2d. These results demonstrate the five P[8] samples are suitable for performing formulation developability experiments (see below), in which the parent protein is expressed with varying levels of truncated, glycosylated and non-native disulfide cross-linked species, and these species are eliminated by molecular engineering to facilitate line-of-sight towards low-cost production as reported elsewhere.^16^

**Fig. 3:**
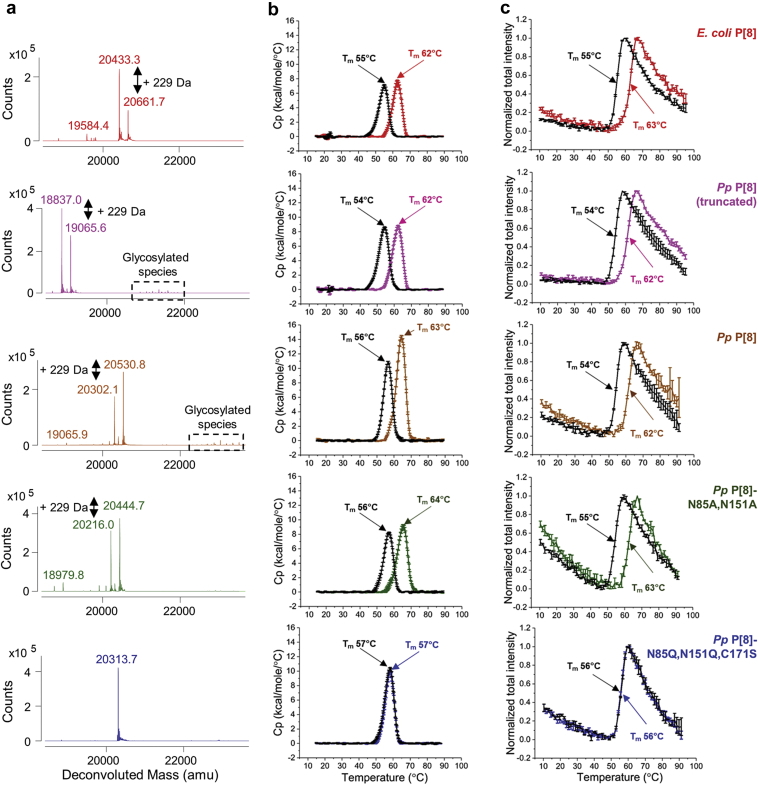
Effect of the vaccine preservative thimerosal on the integrity of the primary structure and conformational stability of NRRV P[8] parent protein and variants as measured by (a) intact protein mass analysis, (b) DSC, and (c) extrinsic fluorescence spectroscopy vs. temperature. For determination of the mean T_m_ values, n = 3 samples were measured with observed 1 SD values of ± −0.1 °C for DSC and ±0.1–0.7 °C for extrinsic fluorescence spectroscopy. Samples were prepared in 10 mM PBS buffer, pH 7.2. See Supplemental Table S1 for summary of T_m_ ±1 SD values.

The compatibility of these five P[8] protein antigens with the vaccine preservative thimerosal was evaluated (Fig. 1). We first compared the molecular composition of the five P[8] protein antigens in the presence and absence of thimerosal by intact protein mass spectrometry (Fig. 3a). The average molecular weight results corresponded to the expected full-length mass of each P[8] variant (within 0.3–0.4 Da mass difference) based on their amino acid sequence. The following additional species were also observed including (1) +131 Da mass increase observed in *E. coli* P[8] protein due to the presence of an additional *N*-terminal Met as expected,^9^ (2) −1465 Da mass decrease observed in *Pp* P[8] (truncated) sample that likely occurred due to the presence of proteolytic enzymes,^17^^,^^18^ and (3) glycosylated species in the *Pp* P[8] and *Pp* P[8] (truncated) samples with a repeating ~162 Da mass difference pattern indicating high-mannose glycoforms (Fig. 3a, dashed box). As expected, these glycosylated species were not observed in the *Pp* P[8]-N85A,N151A and *Pp* P[8]-N85Q,N151Q,C171S samples. Upon addition of thimerosal, a +229 Da mass adduct to P[8] protein species was observed in four of the five samples (Fig. 3a). For the *Pp* P[8]-N85Q,N151Q,C171S sample, no adduct was observed due to the mutation of the single cysteine residue. The nature of the interaction of thimerosal with the NRRV antigen involves a reversible complexation of a degradation byproduct of thimerosal with the single cysteine residue of the NRRV protein as described in detail in a companion paper.^19^

A combination of differential scanning calorimetry (DSC) and extrinsic fluorescence spectroscopy was then used to assess the conformational stability of the five P[8] protein antigens in presence and absence of thimerosal (Fig. 3b and c). A single major endothermic peak with T_m_ values between 62 and 64 °C was observed for four of the P[8] molecules with the *Pp* P[8]-N85Q,N151Q,C171S displaying ~5 °C lower T_m_ value (Fig. 3b, colored traces) by DSC analysis. In presence of thimerosal, however, four of the P[8] molecules displayed ~7–8 °C decrease in T_m_ values (Fig. 3b, black traces), while for the *Pp* P[8]-N85Q,N151Q,C171S, the T_m_ value (57 °C) displayed no change upon addition of thimerosal. Similar results were observed with each P[8] protein by extrinsic fluorescence spectroscopy showing T_m_ values between 62 and 63 °C, except for *Pp* P[8]-N85Q,N151Q,C171S with a T_m_ value of 56 °C (Fig. 3c, colored traces). The addition of thimerosal decreased T_m_ values of each P[8] molecule by ~7–8 °C, except for *Pp* P[8]-N85Q,N151Q,C171S which was not affected by the presence of thimerosal (Fig. 3c, black traces and Supplementary Table S1). As displayed in the upper panel of the summary Table 1, the DSC and extrinsic fluorescence spectroscopy results demonstrate that *Pp* P[8]-N85Q,N151Q,C171S has a lower T_m_ value compared to *Pp* P[8]. However, the conformational stability of the *Pp* P[8]-N85Q,N151Q,C171S is unaffected by the addition of thimerosal while notable destabilization is observed for the other four P[8] samples. Results from similar experiments in the lower panel of Table 1 (and Supplementary Table S1) are presented with various P[4] samples as discussed below. When good correlations are observed between DSC and extrinsic fluorescence spectroscopy results during early stage developability assessments, as was noted in this case study, the latter would be preferable since much less material is required.

**Table 1:** Summary of Differences in T_m_ (Thermal Melting Temperature) Values for Parent Protein and Site-Directed Mutants of NRRV Antigens P[8] and P[4] Produced in *K. phaffii*, with Respect to *E. coli*-Expressed Parent P[8] or P[4] Antigens, Respectively, as Measured by Differential Scanning Calorimetry (DSC) and Extrinsic Fluorescence Spectroscopy.

	NRRV Antigen	Thimerosal Addition (±)	Change in Tm (°C) (vs. *E. coli* Parent Protein)		Change in Tm (°C) (With vs. Without Thimerosal)	
			DSC	Extrinsic Fluorescence Spectroscopy	DSC	Extrinsic Fluorescence Spectroscopy
P[8]	*E. coli* P[8]	–	NA		−7	−8
		+				
	*Pp* P[8] (truncated)	–	0	−1	−8	−8
		+				
	*Pp* P[8]	–	−1	−1	−7	−8
		+				
	*Pp* P[8]-N85A,N151A	–	2	0	−8	−8
		+				
	*Pp* P[8]-N85Q,N151Q,C171S	–	−5	−7	0	0
		+				
P[4]	*E. coli* P[4]-WT	–	NA		−9	−9
		+				
	*Pp* P[4]	–	0	0	−9	−9
		+				
	*Pp* P[4]-C173S	–	−6	−7	0	0
		+				

Samples were prepared at 0.12 mg/mL in 10 mM PBS buffer at pH 7.2. Differences in T_m_ values were determined both in the presence and absence of 0.01% w/v thimerosal. Each sample was measured at n = 3 and differences were determined from averaged values.

See Fig. 3 and Supplemental Table S1 for Tm ±1 SD values.

NA, not applicable.

Finally, the relative solubility of the five P[8] molecules was compared using an ammonium sulfate precipitation assay since protein particle formation was noted previously with freeze-thaw processing of some of the *E. coli* bulk NRRV antigens.^10^ Due to limited material availability, a full curve of ammonium sulfate vs. protein concentration was generated only for *E. coli* P[8] sample initially (Supplementary Fig. S2A), while for the other samples, only three conditions were selected (Supplementary Fig. S2B). This initial analysis showed lower precision due to limited sampling. No major differences between the relative solubility of the five P[8] molecules were observed, albeit there was a trend that the *E. coli* P[8] and *Pp* P[8] (truncated) samples had somewhat higher values (Supplementary Fig. S2B). To further understand these small differences based on limited testing, a full curve was then generated on *Pp* P[8] and *Pp* P[8]-N85Q,N151Q,C171S and compared with the *E. coli* P[8]. The overall curves and the ammonium sulfate midpoint values were similar and ranged between 1.4 and 1.6 M (Supplementary Fig. S2C and S2D). Based on these combined results, we conclude the five P[8] protein antigens have overall similar relative solubility profiles.

### Stage 2 Accelerated and Real-Time Storage Stability Studies of Down-Selected NRRV P[8] Antigens

Based on Stage 1 results described above, two P[8] protein antigens were down-selected including *Pp* P[8]-N85Q,N151Q,C171S, which demonstrated resistance to thimerosal destabilization, and the parent *Pp* P[8] as a control. Additional material (~15–20 mg) was generated for this Stage 2 testing (see Fig. 1), and antibody binding (*in vitro* antigenicity) and DSC (structural integrity) were down-selected as the most informative methods to monitor the storage stability in the presence and absence of the aluminum adjuvant (Alhydrogel®) and preservative (thimerosal). The ELISA format replaced the BLI format to monitor antibody binding due to the presence of the aluminum adjuvant which was incompatible with BLI method (data not shown). The Stage 2 study was setup at different temperatures (4, 25 and 37 °C) over 12 weeks of storage to generate sufficient stability data to compare and rank-order the different samples and formulation conditions.

**Fig. 4:**
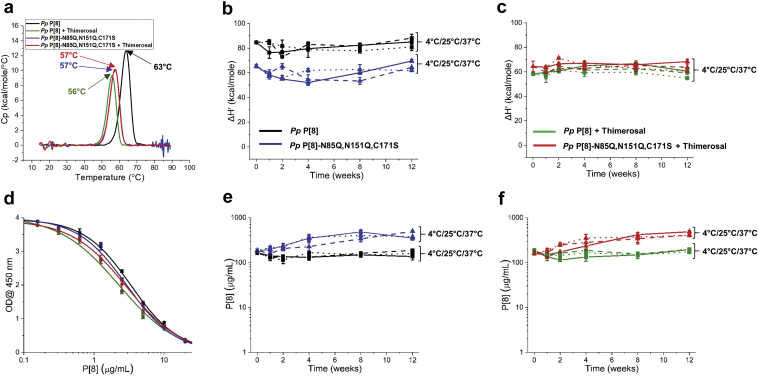
Accelerated and real-time stability study over 12 weeks for NRRV P[8] parent antigen *Pp* P[8] and variant *Pp* P[8]-N85Q,N151Q,C171S in solution. Samples were prepared ± thimerosal at 4 °C (solid lines), 25 °C (dashed lines) and 37 °C (dotted lines) and stability was monitored by DSC and ELISA. (a) Representative DSC thermograms of NRRV proteins in solution at time 0 at 4 °C with indicated T_m_ values. Storage stability profiles of P[8] samples at 4 °C, 25 °C, and 37 °C as measured by DSC (apparent enthalpy values, ΔH′) either (b) without thimerosal, or (c) in the presence of 0.01% w/v thimerosal. (d) Representative ELISA dose response curves at time 0 after storage at 4 °C. Storage stability profiles of P[8] samples at 4 °C, 25 °C, and 37 °C as measured by ELISA either (e) without thimerosal, or (f) in the presence of 0.01% w/v thimerosal. Stability samples contained 0.12 mg/mL protein in 10 mM sodium phosphate, 150 mM NaCl, pH 7.0. Error bars represent mean and range of values with n = 2 (2 sample vials × 1 measurement/vial) for DSC, and mean ± 1 SD for n = 4 (2 sample vials x 2 measurements/vial) for ELISA.

First, the storage stability of *Pp* P[8] and *Pp* P[8]-N85Q,N151Q,C171S in solution (i.e., no aluminum adjuvant) was evaluated in the presence and absence of thimerosal. The conformational stability of *Pp* P[8] and *Pp* P[8]-N85Q,N151Q,C171S over time was monitored by DSC by assessing the change in T_m_ values as well as the change in apparent enthalpy (ΔH′). At time 0, the T_m_ and ΔH′ values for *Pp* P[8] decreased by 7 °C and ~30%, respectively, in the presence (vs. absence) of thimerosal (Fig. 4a). In contrast, *Pp* P[8]-N85Q,N151Q,C171S showed no change in T_m_ or ΔH′ values in the presence or absence of thimerosal (Fig. 4a). During 12 weeks of storage, no changes in the conformational stability, as measured by ΔH’, were observed for *Pp* P[8] or *Pp* P[8]-N85Q,N151Q,C171S in absence (Fig. 4b) or presence (Fig. 4c) of thimerosal at any of the three temperatures. No change in T_m_ values were observed over the course of the stability study (data not shown).

Concomitantly, antibody binding was measured for these same stability samples by a competitive ELISA assay (Fig. 4d). At time 0, the antigenicity of *Pp* P[8] and *Pp* P[8]- N85Q,N151Q,C171S were comparable irrespective of the absence or presence of thimerosal (Fig. 4d). During 12 weeks of storage, however, *Pp* P[8]-N85Q,N151Q,C171S showed a gradual increase in antibody affinity compared to *Pp* P[8] in absence (Fig. 4e) or presence of thimerosal (Fig. 4f). This progressive increase in antibody affinity correlated with time-dependent proteolysis (*N*-terminal truncations in P2 epitope region) observed in *Pp* P[8]-N85Q,N151Q,C171S samples under all storage temperatures in absence or presence of thimerosal (see Supplementary Figs. S3 and S4). Nonetheless, no change in antibody binding was observed for either *Pp* P[8] or *Pp* P[8]-N85Q,N151Q,C171S in solution during the 12 weeks of storage stability study at 4, 25 and 37 °C in absence or presence of thimerosal.

**Fig. 5:**
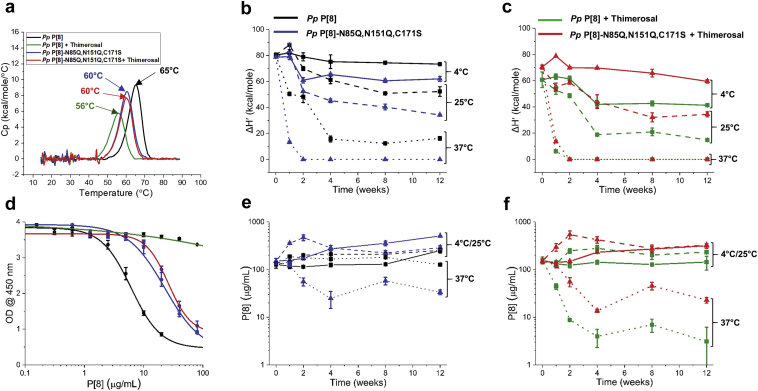
Accelerated and real-time stability study over 12 weeks for Alhydrogel (AH)-adsorbed NRRV P[8] parent antigen *Pp* P[8] and AH-adsorbed variant *Pp* P[8]-N85Q,N151Q,C171S. Samples were prepared ± thimerosal at 4 °C (solid lines), 25 °C (dashed lines) and 37 °C (dotted lines) and stability was monitored by DSC and ELISA. (a) Representative DSC thermograms of AH-adsorbed NRRV P[8] proteins at time 0 at 4 °C with indicated T_m_ values. Storage stability profiles of AH-adsorbed-P[8] samples at 4 °C, 25 °C, and 37 °C as measured by DSC (apparent enthalpy values, ΔH′) either (b) without thimerosal, or (c) in the presence of 0.01% w/v thimerosal. (d) Representative ELISA dose response curves after 12 weeks at 37 °C. Storage stability profiles of AH-adsorbed-P[8] samples at 4 °C, 25 °C, and 37 °C as measured by ELISA either (e) without thimerosal, or (f) in the presence of 0.01% w/v. Stability samples contained 0.12 mg/mL protein, 1.125 mg/mL aluminum adjuvant in 0.5 mM sodium phosphate, 150 mM NaCl, pH 7.0. Error bars represent mean and range of values with n = 2 (2 sample vials × 1 measurement/vial) for DSC, and mean ± 1 SD for n = 4 (2 sample vials × 2 measurements/vial) for ELISA.

Second, a similar 12-week storage stability study with *Pp* P[8] and *Pp* P[8]-N85Q,N151Q,C171S was then performed with the two P[8] antigens adsorbed to an aluminum salt adjuvant (Alhydrogel®, AH) in the presence and absence of thimerosal. At time 0, the addition of thimerosal decreased the conformational stability with respect to T_m_ and ΔH′ values of AH-adsorbed *Pp* P[8] by 9 °C and ~25%, respectively (Fig. 5a) as measured by DSC. No change in T_m_ or ΔH’ values were observed for AH-adsorbed *Pp* P[8]-N85Q,N151Q,C171S in presence vs. absence of thimerosal (Fig. 5a). This time zero result for AH-adsorbed antigens was similar to that observed with the two P[8] proteins in solution as described above.

The stability profiles of the AH-adsorbed P[8] samples, however, were notably different (compared to the in solution counterparts) as measured by DSC. In the absence of thimerosal, AH-adsorbed *Pp* P[8] was more stable than AH-adsorbed *Pp* P[8]-N85Q,N151Q,C171S across the various time points and temperatures (Fig. 5b). For example, after 12 weeks of storage, AH-adsorbed *Pp* P[8]-N85Q,N151Q,C171S showed lower ΔH′ values when stored at 4 °C (~15%) and 25 °C (~35%) compared to AH-adsorbed *Pp* P[8]. At 37 °C, a complete loss of ΔH′ was observed for *Pp* P[8]-N85Q,N151Q,C171S after 2 weeks of storage (Fig. 5b). Interestingly, in the presence of thimerosal, AH-adsorbed *Pp* P[8]-N85Q,N151Q,C171S was more stable than AH-adsorbed *Pp* P[8] (Fig. 5c). For example, *Pp* P[8] showed lower ΔH′ values at 4 °C (~30%) and 25 °C (~55%) compared to *Pp* P[8]-N85Q,N151Q,C171S in presence of thimerosal. However, at 37 °C after 2 weeks, a complete loss of signal (ΔH’ value zero) was observed for both *Pp* P[8] and *Pp* P[8]-N85Q,N151Q,C171S (Fig. 5c) indicating both molecules are labile under such forced degradation conditions. Interestingly, no change in T_m_ values was observed for AH-adsorbed *Pp* P[8] and *Pp* P[8]-N85Q,N151Q,C171S samples over the course of stability study (data not shown).

The same AH-adsorbed P[8] stability samples were also tested for antibody binding (see Fig. 5d for representative ELISA data for selected samples). In the absence of thimerosal, no change in antibody binding was observed for either *Pp* P[8] or *Pp* P[8]-N85Q,N151Q,C171S samples during the 12 weeks of storage at 4 °C or 25 °C (Fig. 5e). After 12 weeks at 37 °C, antibody binding remained unchanged for AH-adsorbed *Pp* P[8], however, for the AH-adsorbed *Pp* P[8]-N85Q,N151Q,C171S, ~60–70% antibody binding was lost in the absence of thimerosal (Fig. 5e). In the presence of thimerosal at 4 or 25 °C, both proteins showed good stability over 12 weeks as measured by antibody binding (Fig. 5f). In contrast, in presence of thimerosal at 37 °C, complete loss of binding was observed with AH-adsorbed *Pp* P[8] (Fig. 5f). However, AH-adsorbed *Pp* P[8]-N85Q,N151Q,C171S remained unaffected by thimerosal addition and displayed same stability profile (Fig. 5e and f). These results confirmed that the stability profile of the *Pp* P[8]-N85Q,N151Q,C171S is not affected by thimerosal addition (see discussion).

### Stage 1 and 2 Testing of a Second NRRV Antigen: P[4] and Variants

To further examine the effects of Alhydrogel and thimerosal on NRRV antigens, both Stage 1 and Stage 2 testing were performed on different variants of a second antigen, NRRV P[4] from the candidate NRRV vaccine. Samples included parent antigen made in *E. coli* (*E. coli* P[4]) and *K. phaffii* (*Pp* P[4]) and one variant *Pp* P[4]-C173S. The complete results from the Stage 1 testing, along with a mechanistic description of thimerosal-induced destabilization of P[4] antigen and its effect on protein's structural integrity and local flexibility (as measured by hydrogen exchange mass spectrometry), is presented in detail in a companion paper.^19^ We summarize here, however, the conformational stability results (see lower panel of Table 1) to better interpret the results from accelerated and real-time stability study (Stage 2) described in this work. The conformational stability of the *E. coli* and *K. phaffii* derived P[4] were determined by DSC and extrinsic fluorescence spectroscopy. In the absence of thimerosal, *E. coli* P[4] and *Pp* P[4] had similar T_m_ values, while in the presence of thimerosal, the T_m_ values decreased by 7–8 °C. The T_m_ value of *Pp* P[4]-C173S was 6–7 °C lower than *E. coli* P[4] and *Pp* P[4], however, it remained unaltered in presence of thimerosal (see lower panel of Table 1). These results demonstrate that *Pp* P[4]-C173S has inherently lower conformational stability than *Pp* P[4], but is resistant to thimerosal-induced destabilization, similar to our observations with P[8] variants described above.

**Fig. 6:**
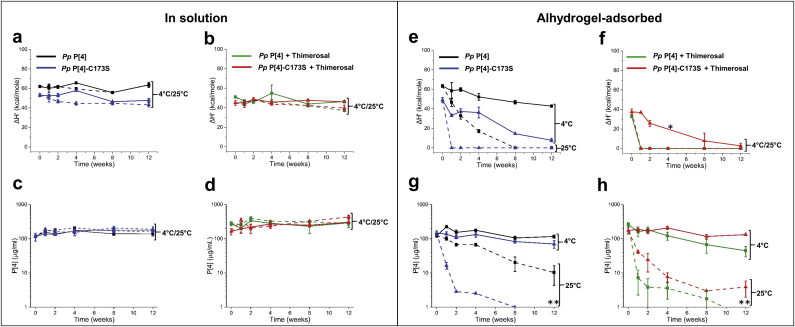
Accelerated and real-time stability study over 12 weeks for NRRV P[4] parent antigen *Pp* P[4] and variant *Pp* P[4]-C173S both in solution and adsorbed to Alhydrogel. Samples were prepared ± thimerosal and stored at 4 °C (solid lines) and 25 °C (dashed lines) and stability was monitored by DSC and ELISA. Storage stability profiles of P[4] samples in solution at 4 °C and 25 °C as measured by DSC (apparent enthalpy values, ΔH′) either (a) without thimerosal, or (b) in the presence of 0.01% w/v thimerosal. Storage stability profiles of P[4] samples in solution at 4 °C and 25 °C as measured by ELISA either (c) without thimerosal, or (d) in the presence of 0.01% w/v thimerosal. Storage stability profiles of AH-adsorbed-P[4] samples in solution at 4 °C and 25 °C as measured by DSC (apparent enthalpy values, ΔH′) either (e) without thimerosal, or (f) in the presence of 0.01% w/v thimerosal. Storage stability profiles of AH-adsorbed P[4] samples in solution at 4 °C and 25 °C as measured by ELISA either (g) without thimerosal, or (h) in the presence of 0.01% w/v. The *Pp* P[4] and *Pp* P[4]-C173S stability samples in solution contained 0.12 mg/mL protein, in 10 mM sodium phosphate, 150 mM NaCl, pH 7.0. The AH-adsorbed *Pp* P[4] and AH-adsorbed *Pp* P[4]-C173S stability samples contained 0.12 mg/mL protein, 1.125 mg/mL aluminum adjuvant in 0.5 mM sodium phosphate, 150 mM NaCl, pH 7.0. Asterisk and double asterisks symbols indicate no measurement and values below 1 μg/mL, respectively. Error bars represent mean and range of values with n = 2 (2 sample vials × 1 measurement/vial) for DSC, and mean ± 1 SD for n = 4 (2 sample vials × 2 measurements/vial) for ELISA.

For the Stage 2 testing with *Pp* P[4] and *Pp* P[4]-C173S, a similar accelerated and real-time stability study (as described above for P[8] samples) was setup at 4 and 25 °C only (no 37 °C), with the goal to determine the effects of adjuvant adsorption (Alhydrogel®) and preservative addition (thimerosal). For the solution samples (no aluminum adjuvant), after 12 weeks of storage at 4 or 25 °C, no loss of conformational stability, as measured by ΔH’ values by DSC, was observed for *Pp* P[4] or *Pp* P[4]-C173S in the absence (Fig. 6a) or presence (Fig. 6b) of thimerosal. In addition, no loss in antibody binding was observed for these two P[4] protein samples in solution in the absence (Fig. 6c) or presence (FIG. 6d) of thimerosal.

For the aluminum-adjuvant adsorbed P[4] samples in the absence of thimerosal, as measured by DSC, the ΔH′ values of the AH-adsorbed *Pp* P[4]-C173S decreased substantially (~85%) after storage for 12 weeks at 4 °C, while for the AH-adsorbed *Pp* P[4], ΔH′ values decreased by ~30% (Fig. 6e). In addition, the rate of decrease in ΔH′ values in both samples was exacerbated when stored at 25 °C, but with similar trends (i.e., the rate of decrease was faster for *Pp* P[4]-C173S). In the presence of thimerosal, AH-adsorbed *Pp* P[4] displayed a complete loss of signal (ΔH′ of zero) at 4 °C upon 1 week of storage only (Fig. 6f). The rate of decrease in ΔH’ values in AH-adsorbed *Pp* P[4]-C173S was much slower with values similar to that in the absence of thimerosal (Fig. 6f).

The AH-adsorbed P[4] stability samples were concomitantly tested for antibody binding. AH-adsorbed *Pp* P[4] showed similar antibody binding (with a trend toward somewhat improved binding) compared to the AH-adsorbed *Pp* P[4]-C173S in absence of thimerosal at 4 °C over 12 weeks of storage (Fig. 6g). At 25 °C in absence of thimerosal, a notable decrease in antibody binding was observed for AH-adsorbed *Pp* P[4]-C173S when compared to AH-adsorbed *Pp* P[4] (Fig. 6g). In contrast, in presence of thimerosal, AH-adsorbed *Pp* P[4] formulation showed ~60% loss in antibody binding while the AH-adsorbed *Pp* P[4]-C173S showed little loss over 12 weeks of storage at 4 °C (Fig. 6h). At 25 °C in the presence of thimerosal, a complete loss in antibody binding was observed for *Pp* P[4] and *Pp* P[4]-C173S after 1 week and beyond of storage (Fig. 6h). In summary, similar to the P[8] variant storage stability results, these P[4] stability studies (i.e., improved storage stability for the AH-adsorbed *Pp* P[4]-C173S compared to AH-adsorbed *Pp* P[4]) demonstrate the removal of the one free cysteine residue in the NRRV protein improves the storage stability of the antigen in presence of thimerosal when adsorbed to aluminum adjuvant.

## Discussion

One key approach for lowering vaccine costs is to reduce their development costs including the chemistry, manufacturing and control (CMC) activities. Developability assessments of small and large molecule drug candidates can help to identify and address issues related to instability, manufacturability, delivery, safety and efficacy of new chemical entities during the early stages of development. Such developability assessments of monoclonal antibody (mAb) candidates includes designing mAbs to have improved pharmaceutical properties including reduced propensity for reversible self-association and aggregation, thus improving solubility and stability while maintaining biological efficacy.^20^ Developability assessments can be considered part of the Quality by Design (QbD) paradigm encouraged by regulatory agencies for drug development.^21^ Although developability assessments have been widely applied to the design of small molecule and monoclonal antibodies (mAbs) drug candidates, such considerations are just beginning as part of antigen design for vaccine development, especially in terms of optimizing vaccine antigens for improved stability as formulated drug products. For example, recent work from our laboratories performed a formulation developability assessment of candidate HIV vaccine antigens^22^; this study, however, required relatively large amounts of material and was focused on a frozen liquid formulation for Phase 1 clinical trials. Here, we have presented a two-step workflow for assessing the suitability of recombinant protein antigens for formulation with adjuvants and preservatives using minimal material. The approach incorporates a strategy to optimize both the antigen by molecular design and the formulation conditions with a focus on improving vaccine stability in dosage forms targeted for use in LMICs (Fig. 1).

In this work, we examined a total of eight NRRV protein antigens including parent protein antigens (made in *E. coli* and *K. phaffii*) as well as site-directed variants of P[8] and P[4] produced in *K. phaffii*. The NRRV variants evaluated here were designed by molecular engineering to enhance P[8] and P[4] manufacturability (i.e., expression levels, molecular properties, immunogenicity) while lowering production costs as described elsewhere.^16^ Using *K. phaffii* as the expression host enables low-cost manufacturing of recombinant protein vaccine antigens via continuous processing^23^ of secreted protein which minimizes cell-based impurities thus reducing costs associated with downstream processing.^24^ *K. phaffii* secreted recombinant proteins have shown >80% purity even before purification.^25^ Challenges using *K. phaffii* as a manufacturing platform include the presence of proteolytic enzymes (resulting in proteolytic degradation of the expressed recombinant protein product^18^) and post-translational modifications via *N*-linked glycosylation^26^ (which may or may not be a desirable trait depending on the protein candidate^27^). The *Pp* P[8]-N85A,N151A and *Pp* P[8]-N85Q,N151Q,C171S variants were generated to prevent these alterations, yet retain expression levels and immunological properties.^16^ The formation of non-native disulfide bonds is a known degradation pathway for NRRV antigens during storage.^9^, ^10^, ^11^ The *Pp* P[4]-C173S and *Pp* P[8]-N85Q,N151Q,C171S variants prevented cysteine-linked dimerization yet retained expression levels and immunological properties.^16^ At the same time, we show in this work that the inherent conformational stability of these targeted variants was lower than the parent antigen (with T_m_ values 5–7 °C lower; see Table 1), yet still showed improved storage stability (compared to parent antigen) in the presence of thimerosal and aluminum adjuvant (see below).

### Stage 1 Developability Assessments of Formulation Variables With NRRV P[8] and P[4] Antigens and Their Variants

We evaluated the effect of two commonly-used vaccine formulation additives including adjuvants (aluminum hydroxide, AH) and preservatives (thimerosal) on the stability of these eight NRRV antigens. Since subunit vaccines generally elicit a weaker immune response than traditional whole-cell preparations,^28^ adjuvants are required to boost their immune responses. Aluminum-salt adjuvants have been used in commercial vaccines for over 80 years resulting in a long, well-established safety profile.^5^^,^^29^ When compared to newer adjuvant technologies^28^ (e.g., oil-in-water emulsions and immune-stimulating molecules), aluminum-salt adjuvants can be produced at lower costs, and thus were the focus of this work. Interestingly, AH-adsorbed Pp P[8] and Pp P[8]-N85Q,N151Q,C171S showed ~2–3 °C higher Tm values than their in-solution counterparts at time zero (Fig. 4, Fig. 5). Such AH-induced stabilization, however, is an antigen-specific phenomenon, and both stabilization and destabilization have been observed with other antigens (and these effects may change over time during storage).^30^, ^31^, ^32^ It would be of interest as part of future work to correlate the extent and strength of NRRV antigen binding to AH to conformational stability measurements by DSC.

Multi-dose formulations offer cost savings over single-dose presentations by minimizing product packaging and wastage, and reducing space requirements in the vaccine cold chain.^33^ Multi-dose formulations typically contain anti-microbial preservatives (APs) to prevent growth of microorganisms that could potentially be introduced during multiple drawings from a single container. APs can, however, cause vaccine antigen instability by inducing alterations in protein structure, leading to aggregation^34^^,^^35^ and loss of potency.^36^^,^^37^ Thimerosal is widely used as a vaccine preservative, especially in the childhood pentavalent vaccine given in LMICs,^38^ and was a focus in this work. Previous studies have shown that thimerosal decomposes to thiosalicylate and ethylmercury in solution, with the latter able to form coordinate bonds with free thiol groups in proteins.^39^ Consistent with the literature, a +229 Da mass adduct was observed by intact protein mass spectrometry for P[8] parent and mutant proteins containing the one cysteine residue (Fig. 3a). The adduct was not observed in *Pp* P[8]-N85Q,N151Q,C171S, which further supports the formation of ethylmercury-P[8] adduct species through the single cysteine residue present in native P[8] protein (Fig. 3a). The molecular mechanism of the interaction of thimerosal with P[4] antigen, along with its effects of protein structural integrity, including antibody binding and flexibility (as measured by HX-MS), is described in a companion paper.^19^

The results from Stage 1 of the developability assessment suggest *Pp* P[8]-N85Q,N151Q,C171S has improved compatibility with thimerosal thereby potentially enabling a multi-dose formulation. Given the ~5–7 °C lower inherent conformational stability of *Pp* P[8]-N85Q,N151Q,C171S, however, the relative storage stability of fully formulated *Pp* P[8]-N85Q,N151Q,C171S was difficult to predict. A similar situation was observed for the *Pp* P[4]-C173S compared to the *Pp* P[4]. To better understand the possible correlations of Stage 1 testing results with Stage 2 storage stability profiles as formulated vaccine (with adjuvant and preservative), two NRRV P[8] antigens (*Pp* P[8], *Pp* P[8]-N85Q,N151Q,C171S) and two NRRV P[4] antigens (*Pp* P[4], and *Pp* P[4]-C173S) were expressed and purified in larger amounts (15–20 mg) and evaluated in a 12-week accelerated and real-time storage stability study as described below.

### Stage 2 Storage Stability Studies With Down-Selected NRRV P[8] and P[4] Antigens and Their Variants Both in the Presence and Absence of Alhydrogel and Thimerosal

The 12-week storage stability profile at different temperatures of the four AH-adsorbed NRRV antigens (*Pp* P[8] vs. *Pp* P[8]-N85Q,N151Q,C171S, and *Pp* P[4] vs. *Pp* P[4]-C173S) was evaluated by DSC (structural integrity) and ELISA (antibody binding). Interestingly, the rank-ordering of the storage stability results of the AH-adsorbed antigens correlated well with the Stage 1 developability results. For example, the AH-bound *Pp* P[8] showed better storage stability than the AH-bound *Pp* P[8]-N85Q,N151Q,C171S in the absence of thimerosal, but the opposite trend was observed in the presence of thimerosal (Fig. 5). By comparison, during Stage 1 testing, *Pp* P[8] showed improved conformational stability compared to the *Pp* P[8]-N85Q,N151Q,C171S (T_m_ values were 5–7 °C higher) in the absence of thimerosal. Upon thimerosal addition, however, *Pp* P[8] was destabilized by 7–8 °C while *Pp* P[8]-N85Q,N151Q,C171S was not affected. Similar trends were observed with the two AH-adsorbed P[4] NRRV antigens, AH-bound *Pp* P[4] and AH-bound *Pp* P[4]-C173S during storage (Fig. 6) and during Stage 1 testing. These results reinforced the predictability of Stage 1 developability assessment data sets to rank order the storage stability of AH-adjuvanted formulations of NRRV P[8] and P[4] antigens (and their site-directed mutants) in the presence and absence of thimerosal.

The trends in AH-adsorbed NRRV antigen stability over the 12-week storage stability study as measured by ELISA (antibody binding) showed a qualitative agreement with the trends measured by DSC (structural stability), though some exceptions were noted. For example with P[8] antigens, DSC measurements of AH-bound *Pp* P[8]-N85Q,N151Q,C171S (±thimerosal) indicated almost a complete loss of signal after 1 week at 37 °C (Fig. 5b and c), however, ELISA measurements displayed notable antibody binding after 2 weeks at 37 °C (Fig. 5e and f). Similarly with P[4] antigens, DSC measurements of AH-bound *Pp* P[4]-C173S (+thimerosal) indicated essentially a total loss of signal after 1 week at 25 °C (Fig. 6f), however, ELISA measurements indicated antibody binding remained after ~4 weeks at 25 °C (Fig. 6h). This lack of agreement between DSC and ELISA measurements is not unexpected and can be attributed to the two assays reporting on different structural features of the protein antigen. For instance, NRRV could partially unfold and result in decreased ΔH’ value as measured by DSC, however, such structural alterations can occur without perturbing the antibody binding epitope, thus not affecting the antibody binding measured by ELISA.

Interestingly, no such correlations of Stage 1 developability data vs. Stage 2 storage stability were observed for the NRRV antigens formulated in solution (i.e., no Alhydrogel®). The four NRRV antigens stored in solution were stable across the various temperatures over the course of the 12-week study, as measured by both DSC and ELISA, regardless of the presence or absence of thimerosal (Fig. 4, Fig. 6). Although it is possible such a correlation may emerge during longer term stability studies (e.g., 2 years), such studies were outside the scope of this work. Over the course of these 12-week stability studies, we speculate that the adsorption of the NRRV antigens to the surface of the aluminum adjuvant likely acts as a “catalyst” for accelerating irreversible conformational changes in the protein antigen. For example, a progressive decrease of ΔH’ values were observed during storage of the AH-bound P[8] and AH-bound P[4] antigens (Fig. 5, Fig. 6, respectively), but this was not seen with same antigens in solution over the course of the 12-week stability study (Fig. 4, Fig. 6).

It is possible that the impact of lower inherent conformational stability (i.e., lower T_m_ values) of a protein antigen on long-term storage stability is evident only in the presence of an additional destabilization force such as surface adsorption. For example, addition of thimerosal and/or mutation of cysteine residue could induce a structural alteration in NRRV antigen. However, these effects may be reversible in nature in solution, and do not affect the storage stability of the antigen (over the time course examined in this work). In fact, as described in a companion paper,^19^ increased structural flexibility of the parent P[4] antigen is noted in the presence of thimerosal. In contrast, when adsorbed to a surface such as an aluminum adjuvant, such structural alterations of a protein antigen become irreversible in nature since exposure of buried amino acid residues (due to increased flexibility) can lead to interactions with the aluminum surface triggering the irreversible structural changes of the protein. In fact, previous studies have reported the destabilizing effects of aluminum adjuvant adsorption on protein conformational stability using a series of model proteins as well as with recombinant botulinum neurotoxin and hepatitis B vaccine antigen.^32^^,^^40^, ^41^, ^42^

## Conclusions

In summary, a two-step developability assessment workflow is presented for screening variants of recombinant protein vaccine antigens using small amounts of material. The goal was to rapidly formulate new recombinant protein vaccine antigens as low-cost, aluminum adjuvanted, multi-dose vaccines targeted for use in LMICs. We applied this formulation developability workflow to a series of variants of two NRRV antigens, P[8] and P[4], produced in *E. coli* and a low cost vaccine platform using *K. phaffii.* Stage 1 of this workflow guided protein-design efforts to identify NRRV antigen variants with improved compatibility with preservatives, thereby incorporating a QbD antigen design approach to multi-dose formulation development. Interestingly, these Stage 1 developability results were observed to be predictive of rank-ordering the storage stability of Alhydrogel-adsorbed P[8] and P[4] variants (Stage 2 testing), but were less predictive of the storage stability profiles of solution formulations (no aluminum adjuvant). Such correlations between Stage 1 and Stage 2 results in this formulation developability workflow can be used to guide and accelerate subsequent rounds of formulation design including, for example, (1) additional rounds of antigen design, (2) screening of stabilizing excipients as well as other preservatives and adjuvants, and (3) compatibility with other vaccine antigens for use in combination vaccines.
