# Erratum to “Rapid developability assessments to formulate recombinant protein antigens as stable, low-cost, multi-dose vaccine candidates: Case-study with non-replicating rotavirus (NRRV) vaccine antigens” [J Pharm Sci 110 (2021) 1042–1053]

**DOI:** 10.1016/j.xphs.2021.03.003

**Published:** 2021-04

This paper was miscategorized in the March 2021 issue and should have been included under the subject category of **Pharmaceutical Biotechnology** with the companion article, “Mechanism of Thimerosal-Induced Structural Destabilization of a Recombinant Rotavirus P[4] Protein Antigen Formulated as a Multi-Dose Vaccine” [J Pharm Sci 110 (2021) 1054–1066].

