# Supplementary material for: Rapid Developability Assessments to Formulate Recombinant Protein Antigens as Stable, Low-Cost, Multi-Dose Vaccine Candidates: Case-Study With Non-Replicating Rotavirus (NRRV) Vaccine Antigens
Source: J Pharm Sci. 2021 Mar;110(3):1042–53. doi: 10.1016/j.xphs.2020.11.039 (PMC7884052; doi:10.1016/j.xphs.2020.11.039)

**Supplemental Information**

**Rapid developability assessments to formulate recombinant protein antigens as stable, low-cost, multi-dose vaccine candidates: Case-study with non-replicating rotavirus (NRRV) vaccine antigens**

Nishant Sawant ^a,d^, Kawaljit Kaur ^a,d^, David A. Holland ^a^, John M. Hickey ^a^, Sanjeev Agarwal ^a,e^, Joseph R. Brady ^b^, Neil C. Dalvie ^b^, Mary Kate Tracey ^b^, M. Lourdes Velez-Suberbie ^c^,

Stephen A. Morris ^c^, Shaleem I. Jacob ^c,f^, Daniel G. Bracewell ^c^, Tarit K. Mukhopadhyay ^c,g^, Kerry R. Love ^b^, J. Christopher Love ^b^, Sangeeta B. Joshi ^a^, and David B. Volkin ^a*^

^a^ Department of Pharmaceutical Chemistry, Vaccine Analytics and Formulation Center, University of Kansas, 2030 Becker Drive, Lawrence, KS 66047, USA

^b^ Department of Chemical Engineering, Koch Institute for Integrative Cancer Research, Massachusetts Institute of Technology, Cambridge, MA 02139, USA

^c^ Department of Biochemical Engineering, University College London, Bernard Katz Building, Gower Street, London WC1E 6BT, UK

***** Corresponding author. Multidisciplinary Research Building, 2030 Becker Drive, Lawrence, KS 66047, USA. *E-mail address:* [volkin@ku.edu](mailto:volkin@ku.edu) (D.B. Volkin).

^d^ These authors contributed equally to this work.

^e^ Current address: Amgen, Thousand Oaks, CA 91320, USA.

^f^ Current address: Oxford BioMedica plc, Oxford, OX4 6LT, UK.

^g^ Current address: Merck & Co., West Point, PA 19486, USA.

**Supplementary Methods**

**Generation of P[4] and P[8] variants in *K. phaffii* (*P. pastoris*): Fed-batch fermentation and purification**

Inocula from working cell banks were grown in BMGY medium incubated at 30°C for fermentations in Basal Salt Medium (BSM)^1^ or 25°C for fermentations in Rich Defined Medium (RDM)^2^, at 250 rpm until they reached an OD_600_ value of ~10. Fermentations were carried out using an ambr^®^ 250 modular microbial system (Sartorius Stedim Biotech, Royston, UK). Please refer to Velez-Suberbie *et al.*^3^ for detailed protocol on expression and purification of P[4] antigen. Fermentations for *K. phaffii* expressing P[8] parent protein that resulted in truncated product (*Pp* P[8] truncated) were carried out using BSM following Invitrogen’s protocol for Mut^+^ cells. Fermenter operating conditions were 30°C, 30% DO, pH 5.00 ± 0.15 (controlled with 10% (v/v) ammonium hydroxide) and antifoam (polypropylene glycol 2000), which was automatically added by the system when required. Fermentations for all other constructs used in the study were carried out using RDM (25°C, 25% DO, pH 6.50 ± 0.15), pH and foaming were controlled as in fermentations in BSM. A pH pulse was implemented at the end of methanol adaptation (~30 hr), pH was reduced to 3.00 ± 0.15 held low for ~4 hr and then ramped for an hr to the higher set point and harvest was done 3 hr after end of pH pulse. The fermentations of *K. phaffii* expressing P[8] parent protein that resulted in full-length product (*Pp* P[8]), and *K. phaffii* expressing P[8] variants (*Pp* P[8]-N85A, N151A, and *Pp* P[8]-N85Q, N151Q,C171S) were carried out at constant pH of 6.5. Methanol adaptation was done in 4 to 5, 10 % incremental steps programmed into the ambr250 software and a range of methanol feed rates were used 7.6 – 20 mL/h per liter of initial fermentation volume. Cell broth was centrifuged at 10,000 x g for 15 min, the supernatant was filtered using 0.80 µm cellulose nitrate and 0.45 µm polyvinylidene difluoride (PVDF) membrane filters (GE Healthcare Life Sciences, Buckinghamshire, UK) and aliquoted into 20 or 30 mL samples and stored at -20°C until further processing.

*Pp* P[8] (truncated) was purified via HIC as follows: 25 mL of clarified fermentation medium was buffer exchanged into 50 mM Tris pH 7.5 by diafiltration at 4°C using a 3 kDa cutoff regenerated cellulose membrane (Amicon Ultra 15, Merck Millipore, Watford, UK). This was accomplished by repeated dilution and re-concentration until the buffer exchange was >99.5%. This was then brought to a final ammonium sulfate concentration of 1 M by addition of a 3 M ammonium sulfate in 50 mM Tris, pH 7.5 stock solution. Material was filtered through a 0.22 µm PVDF membrane filter (Merck Millipore). HIC chromatography was performed via an AKTA Avant system using a 1 mL HiTrap Capto Phenyl (High Sub) column at a flow rate of 1 mL/min. Binding was carried out in 1 M ammonium sulfate_,_ 50 mM Tris, pH 7.5. Bound protein was eluted by step-wise decreases in ammonium sulfate concentration (0.2 M per step), 5 CV per step, with elution occurring at 0.4 M ammonium sulfate. A 2-stage chromatography procedure was applied to purify all of the other variants of P[8] (*Pp* P[8], *Pp* P[8]-N85A,N151A, and *Pp* P[8]-N85Q,N151Q,C171S) as follows: Between 25 – 100 mL of clarified fermentation medium was brought to pH 4.0 by addition of 0.1 M citric acid. Conductivity was then adjusted to 10 mS/cm by addition of 50 mM sodium citrate, pH 4.0 buffer. All material was filtered through a 0.22 µm PVDF membrane filter. This allowed for direct binding onto a 5 mL HiTrap Capto S ImpAct column pre –equilibrated in 50 mM sodium citrate, pH 4.0 buffer. Host cell proteins (HCP) were removed by a wash of 0.2 M NaCl in 50 mM sodium citrate, pH 4.0. Following removal of NaCl, a step of 50 mM Tris pH 7.5 was employed to elute NRRV. This eluate was then applied directly to a 5 mL Capto Q ImpRes column pre-equilibrated in 50 mM Tris, pH 7.5. P[8] was recovered in the flow-through, with remaining HCP and DNA binding to the column. Both columns were operated at a flow rate of 5 mL/min on an AKTA Avant system. All wash and elution steps were 5 CVs.

**Purification of P[4] and P[8] from *E. coli***

Cell pellets of *E. coli* expressing P[4] and P[8] were provided by Dr. Stan Cryz at PATH Vaccine Solutions, Seattle, USA. Purified *E. coli* P[4] and P[8] were produced via a combination of anion exchange – hydrophobic interaction (HIC) – anion exchange chromatography as previously described.^4^

**Sample Preparation for Stage 1 and Stage 2 Studies**

For developability studies (Stage 1), the purified P[8] proteins were dialyzed against 10 mM sodium phosphate, 150 mM NaCl, pH 7.2 (PBS) using 3.5 kDa molecular weight cutoff Slide-A-Lyzer Mini Dialysis devices at 4°C. Protein concentration was determined by UV-visible spectrophotometry as described below. Samples were prepared at 0.12 mg/mL by diluting the dialyzed protein stock solutions with 10 mM PBS buffer. A 200X thimerosal stock solution was prepared in water. Samples containing thimerosal were prepared by mixing with 200X thimerosal stock solution (2% w/v in water) to a final thimerosal concentration of 0.01% w/v. The pH values (7.2 ± 0.2) of samples were verified using an Accumet^®^ XL 25 dual channel pH meter (Fisher Scientific, Hampton, NH).

For accelerated and real-time storage stability studies (Stage 2), *Pp* P[8], *Pp* P[8]-N85Q,N151Q,C171S, *Pp* P[4], and *Pp* P[4]-C173S were dialyzed against 0.5 mM sodium phosphate, 150 mM NaCl, pH 7.0 (0.5 mM PBS) using 3.5 kDa molecular weight cutoff Slide-A-Lyzer Dialysis Cassette at 4°C. The protein concentration was determined by UV-visible spectrophotometry as described below and samples were stored at -80°C. A 2.25 mg/mL aluminum suspension was prepared by diluting 2% Alhydrogel® (10 mg/ml aluminum content) with water and 10X buffer solution (5 mM sodium phosphate, 1.5 M NaCl, pH 7.0) to achieve a target concentration of 0.5 mM phosphate and 150 mM NaCl. To prepare alhydrogel (AH)-adsorbed stability samples, frozen NRRV antigen samples were thawed at room temperature, and equal volumes of 0.24 mg/mL NRRV protein and 2.25 mg/mL aluminum Alhydrogel® solution were mixed. The formulations were kept in a refrigerator overnight for adsorption of antigen to aluminum adjuvant. Adjuvant placebo formulations were prepared similarly using 0.5 mM PBS in place of the protein solution. To prepare non-adjuvanted (in solution) stability samples, the phosphate concentration of the undiluted protein samples was adjusted using an appropriate volume of 1M phosphate, pH 7.0 such that the final phosphate concentration was 10 mM. The phosphate adjusted protein samples were then diluted to 0.12 mg/mL with 10 mM PBS, pH 7.0. Placebo formulations consisted of 10 mM PBS pH 7.0. Thimerosal containing formulations were prepared by adding an appropriate volume of 4% w/v solution of thimerosal in water, such that the final concentration of thimerosal in samples was 0.01% w/v. The final pH values (7.0 ± 0.2) was achieved by adjusting with NaOH/HCl and monitored using an Accumet^®^ XL 25 dual channel pH meter. The formulations were prepared, vialed and capped within a Class II Biosafety Cabinet (Labconco Corporation, Kansas City, MO). These formulations were stored in sealed 2 mL glass vials with 13 mm rubber stoppers (West Pharmaceutical Services, West Whiteland Township, PA), and then stored at 4, 25 and 37°C incubator for up to 12 weeks.

**UV-visible spectroscopy**

The UV-visible absorption spectra of various P[8] and P[4] molecules were recorded with an Agilent-8453 UV-visible spectrophotometer equipped with deuterium (D_2_) and tungsten (W) lamps. The Beer-Lambert law was used to calculate protein concentration based on calculated extinction coefficient 1.73 mg/mL^-1^cm^-1^ for P[8] and 1.65 mg/mL^-1^cm^-1^ for P[4] (calculated using protein sequence using <https://web.expasy.org/protparam/>). All UV-Spectra were corrected for light scattering using a technique included in the manufacturer’s data analysis software (Chemstation UV-Vis analysis software; Agilent Technologies). Please refer to our recent work on physicochemical characterization of NRRV protein antigens for detailed protocol on UV-visible spectroscopy.^5^

**SDS-PAGE**

For reduced SDS-PAGE, samples were mixed with NOVEX^®^ Tricine-SDS sample buffer (1X), 10 mM dithiothreitol and 20 mM iodoacetamide. For non-reduced SDS-PAGE, samples were mixed with NOVEX^®^ Tricine-SDS sample buffer (2X) and 20 mM iodoacetamide. Both reduced and non-reduced SDS-PAGE samples were incubated at room temperature in dark for 30 min. Samples were heated at 90°C for 10 min. Approximately 2 µg of each sample was resolved using NOVEX^™^ 16% Tricine gel with NOVEX^®^ Tricine-SDS as running buffer (1X). Electrophoresis was carried out at 120 V for 110 min. Coomassie Blue R-250 solution (Teknova, Hollister, CA) was used to visualize the protein bands. Gels were destained using a mixture of 10% acetic acid, 40% methanol and 50% water for 2 hr and then overnight with water. Gels were digitized using AlphaImager (Protein Simple, San Jose, CA) gel imaging system.

**Intact protein mass spectrometry**

P[8] samples with and without thimerosal (prepared as described above) were centrifuged at 14,000 × g for 1 min and 80% of the supernatant was transferred to an HPLC vial for intact protein mass analysis. The protein from AH-adsorbed storage stability samples was desorbed using 200 mM phosphate prior to intact protein mass analysis. Mobile phase A consisted of water with 0.1% formic acid, and mobile phase B was acetonitrile with 0.1% formic acid. About 20 pmol of each P[8] sample was injected into an 1220 series LC system (Agilent Technologies), bound to a ZORBAX 300SB C3 column (Agilent Technologies), desalted, and subjected to electrospray ionization time-of-flight mass spectrometry (model 6230B, Agilent Technologies). The LC gradient consisted of 20-70% B over 1 min at a flow rate of 1.5 mL/min. Elution of proteins was monitored using the absorbance signal at 214 nm. 50 µL of isopropanol was injected after each sample to control sample carry-over. The typical electrospray ionization parameters consisted of 290°C gas temperature, 4000 V Vcap, 2000 V nozzle, and 275 V fragmentor voltage. Mass spectra were collected from 700-2800 m/z at a scan rate of 1 spectra/sec. MS spectra were processed using MassHunter Qualitative Analysis software (v B.07.00, Agilent Technologies) with deconvolution range of 10-50 kDa, using 1 Da mass step.

**Differential scanning calorimetry (DSC)**

Differential scanning calorimetry (DSC) analysis was performed using MicroCal VP-Capillary calorimeter (Malvern, UK) equipped with tantalum sample and reference cells. Samples were loaded into temperature-controlled auto sampler tray held at 4°C. Scans were completed from 10 to 90°C using a scanning rate of 60°C/h. Data analysis was performed using the DSC plug-in for the Origin 7.0 software package. After performing reference subtraction and concentration normalization, results were fitted to a ‘Non-2 state’ model with one transition to calculate the melting temperature (T_m_) value. The area under the curve (apparent enthalpy, ΔH’) was calculated using peak integration function in Origin 9.4 software package.

**Extrinsic fluorescence spectroscopy**

A fluorescence plate reader^6^ (Fluorescence Innovations Inc., Minneapolis, MN) was used to measure time resolved fluorescence (TRF). 8-Anilino-1-naphthalene sulfonate (ANS) was used as an extrinsic fluorescence probe. A dye to protein molar ratio of 25:1 was used for sample preparation. The excitation laser (a combo laser) was set at 350 nm. A 405 nm long-pass dichroic mirror, a band pass filter (485 ± 20 nm) and photomultiplier tube (PMT) were used to monitor TRF. The PMT voltage was set at 500 V. Measurements were performed using integration time of 500 ms. Samples were subjected to a 10 to 90°C temperature ramp at a rate of 1.25°C/min. The TRF mode records fluorescence decay waveforms.^6^ Total intensity data (the peak area under the curve for a waveform) at various temperatures was obtained from instrument. Using Origin 9.4 software package, first derivative of total intensity data was plotted against corresponding temperatures to calculate T_m_ value.

**Ammonium sulfate precipitation assay**

A series of test samples (n = 3) were generated by mixing appropriate volumes of P[8] in 10 mM PBS pH 7.2, 3.5 M ammonium sulfate in 10 mM PBS pH 7.2 (3.5 M NH_4_SO_4_) and 10 mM PBS pH 7.2, such that the concentration of P[8] was held constant at 0.2 mg/mL and the concentration of ammonium sulfate ranged from 0 to 2.5 M. A series of blank samples (n = 2) were generated by mixing appropriate volumes of 3.5 M NH_4_SO_4_ and 10 mM PBS pH 7.2, such that the ammonium sulfate concentration matches the concentration in test samples. A standard curve (n = 2) was generated by diluting an appropriate volume of P[8] with 10 mM PBS, pH 7.2, such that the protein concentration ranged from 0.025 – 0.25 mg/mL. The test samples, blanks and standard curve samples were incubated at room temperature for 2 hrs. After 2 hrs, the samples were transferred to a 96-well polystyrene filter plate (Corning #3504; Corning Life Sciences, Corning, NY) which was attached on top of a clear 96-well collection plate (Greiner Bio-One #655001; Greiner Bio-One North America Inc., Monroe, NC). The plates were centrifuged at 4,000 rpm for 30 min. Thereafter, 150 µL of filtrate was transferred into a 96-well UV Star microplate (Greiner Bio-One #655801). The filtrate was measured on a SpectraMax M5 UV-Visible plate reader (Molecular devices, CA) at 280 nm. The protein concentration was determined from the blank subtracted absorbance value using the standard curve.

The remaining P[8] concentration as a function of ammonium sulfate concentrations was fitted into a Boltzmann sigmoidal curve function (Eq. 1)^7^ using Origin 9.4 software package:

| 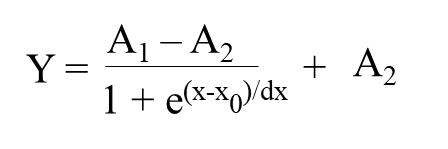 | (1) |
| --- | --- |

where A_1_ = initial absorbance value, A_2_ = final absorbance value, X_0_ = ammonium sulfate midpoint value and dx is the width value which is related to the slope, where a smaller dx value indicates a more steep curve. The ammonium sulfate midpoint value is inversely proportional to the solubility of the protein and is therefore used to compare the relative solubility of P[8] variants. For a 3-point ammonium sulfate precipitation assay, same procedure was followed as described above, except only 3-points (ammonium sulfate midpoint, one point above and below ammonium sulfate midpoint) were used for sample preparation, data interpretation and relative solubility comparison between samples.

**Immunochemical assay (ELISA)**

The details of the inhibition ELISA assay used in this work, including the antibodies used and the nature of their interaction with NRRV antigens, is described elsewhere (McAdams *et al*., manuscript in preparation). Briefly, all test NRRV antigen samples (in solution or AH-adsorbed) were first incubated with a blocking buffer, then serial dilutions were made followed by incubation with a fixed amount of NRRV antigen specific primary antibody overnight on a microplate shaker at room temperature. Corresponding blank samples were prepared using blocking buffer alone, while the saturated samples contained the primary antibody. Samples were centrifuged the next day and the supernatant containing free antibody was transferred to a 96 well plate coated with *E. coli* NRRV P[8]/P[4] standard. The plate was incubated for two hours at 25°C, washed, and the amount of bound primary antibody on the plate was detected with a horseradish peroxidase labeled secondary antibody using a tetramethylbenzidine substrate. The apparent concentration of the NRRV antigen in test samples was determined from the OD_450_ values using the parameters obtained from a 4-point logistic fit of the standard run on each plate.

**References for supplemental section**

1. Invitrogen. 2002. Pichia fermentation process guidelines. (version B053002). Available at <http://tools.thermofisher.com/content/sfs/manuals/pichiaferm_prot.pdf>. Accessed January 12, 2020.

2. Matthews CB, Kuo A, Love KR, Love JC 2018. Development of a general defined

medium for Pichia pastoris. Biotechnology and bioengineering 115(1):103-113.

3. Velez‐Suberbie ML, Morris SA, Kaur K, Hickey JM, Joshi SB, Volkin DB, Bracewell DG, Mukhopadhyay TK 2020. Holistic process development to mitigate proteolysis of a subunit rotavirus vaccine candidate produced in Pichia pastoris by means of an acid pH pulse during fed‐batch fermentation. Biotechnology Progress:e2966.

4. Fix AD, Harro C, McNeal M, Dally L, Flores J, Robertson G, Boslego JW, Cryz S 2015. Safety and immunogenicity of a parenterally administered rotavirus VP8 subunit vaccine in healthy adults. Vaccine 33(31):3766-3772.

5. Agarwal S, Hickey JM, Sahni N, Toth IV RT, Robertson GA, Sitrin R, Cryz S, Joshi SB,Volkin DB 2020. Recombinant subunit rotavirus trivalent vaccine candidate: physicochemical comparisons and stability evaluations of three protein antigens. Journal of pharmaceutical sciences 109(1):380-393.

6. Wei Y, Larson NR, Angalakurthi SK, Russell Middaugh C 2018. Improved fluorescence methods for high-throughput protein formulation screening. SLAS TECHNOLOGY: Translating Life Sciences Innovation 23(6):516-528.

7. Yamniuk AP, Ditto N, Patel M, Dai J, Sejwal P, Stetsko P, Doyle ML 2013. Application of a kosmotrope-based solubility assay to multiple protein therapeutic classes indicates broad use as a high-throughput screen for protein therapeutic aggregation propensity. Journal of pharmaceutical sciences 102(8):2424-2439.

**Supplementary Table S1.** Summary of T_m_ (thermal melting temperature) values for parent protein and site-directed mutants of NRRV antigens P[8] and P[4] produced in *K. phaffii*, as measured by differential scanning calorimetry (DSC) and extrinsic fluorescence spectroscopy. Samples were prepared at 0.12 mg/mL in 10 mM PBS buffer at pH 7.2. T_m_ values were determined both in the presence and absence of 0.01% w/v thimerosal. Each sample was measured at n = 3 with average values reported in the table. SD values ranged from 0.1-0.7 for all samples.

| **NRRV Antigen** | | **T_m_ (°C)**  **(DSC)** | | **T_m_ (°C) (Extrinsic fluorescence spectroscopy )** | |
| --- | --- | --- | --- | --- | --- |
|  |  | **Thimerosal Addition (-/+)** | | **Thimerosal Addition (-/+)** | |
|  |  | **-** | **+** | **-** | **+** |
| P[8] | *E. coli* P[8] | 62 | 55 | 63 | 55 |
|  | *Pp* P[8] (truncated) | 62 | 54 | 62 | 54 |
|  | *Pp* P[8] | 63 | 56 | 62 | 54 |
|  | *Pp* P[8]-N85A,N151A | 64 | 56 | 63 | 55 |
|  | *Pp* P[8]-N85Q,N151Q,C171S | 57 | 57 | 56 | 56 |
|  |  |  |  |  |  |
| P[4] | *E. coli* P[4]-WT | 56 | 47 | 55 | 46 |
|  | *Pp* P[4] | 56 | 47 | 55 | 46 |
|  | *Pp* P[4]-C173S | 50 | 50 | 48 | 48 |

**Supplementary Figures**

**Supplementary Figure S1.** Reduced SDS-PAGE analysis of indicated P[8] samples with and without Endo-H treatment. MW markers indicate kDa values of samples. Bands associated with Endo H itself and glycosylated P[8] are shown by arrows.


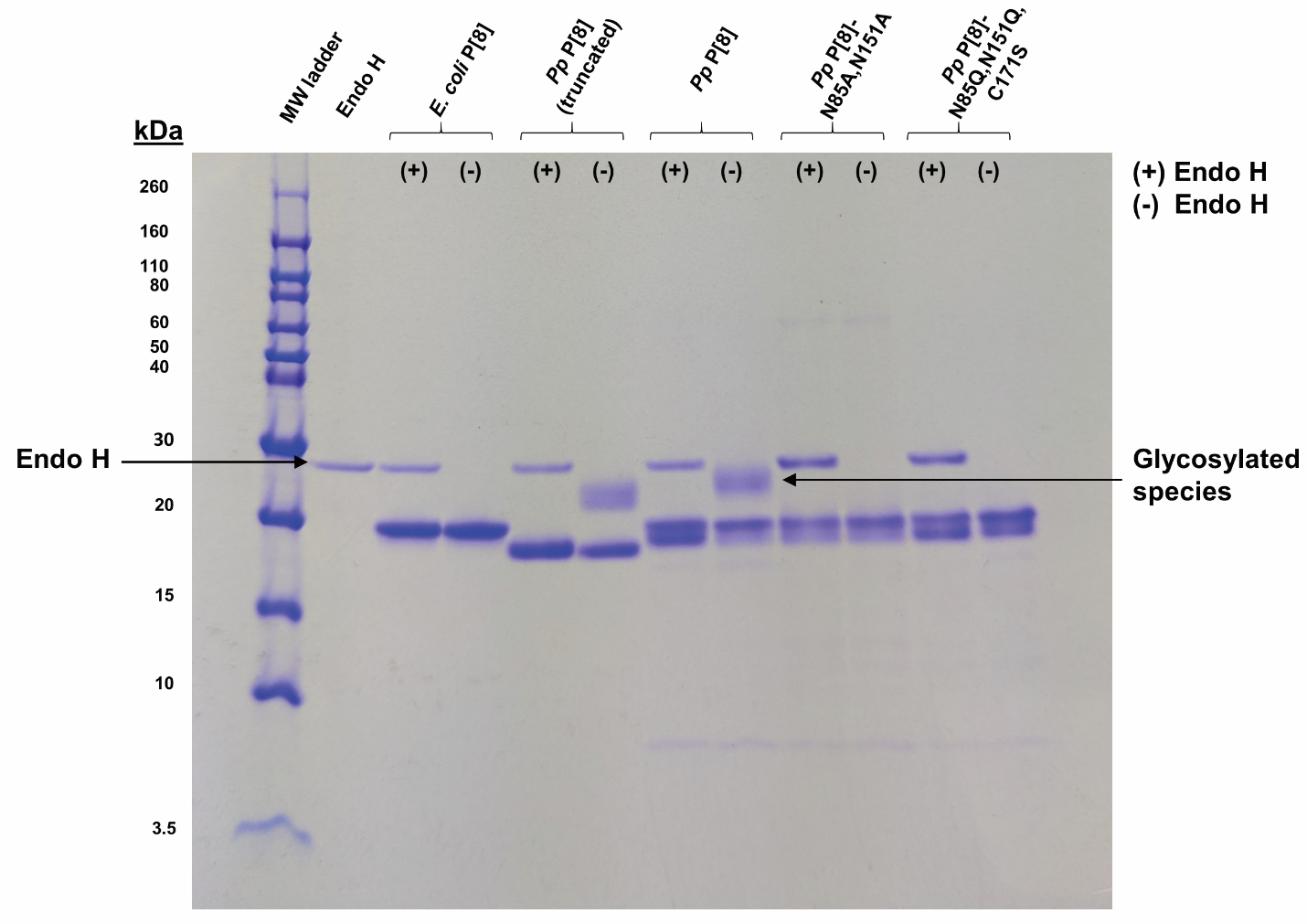


**Supplementary Figure S2.** Relative solubility assessment for various P[8] samples using ammonium sulfate precipitation assay. (A) Solubility curve for *E. coli* P[8], (B) 3-point solubility assessment for various P[8] samples, (C) Solubility curve for *Pp* P[8], and (D) Solubility curve for *Pp* P[8]-N85Q,N151Q,C171S. (n=3, 1SD)

**
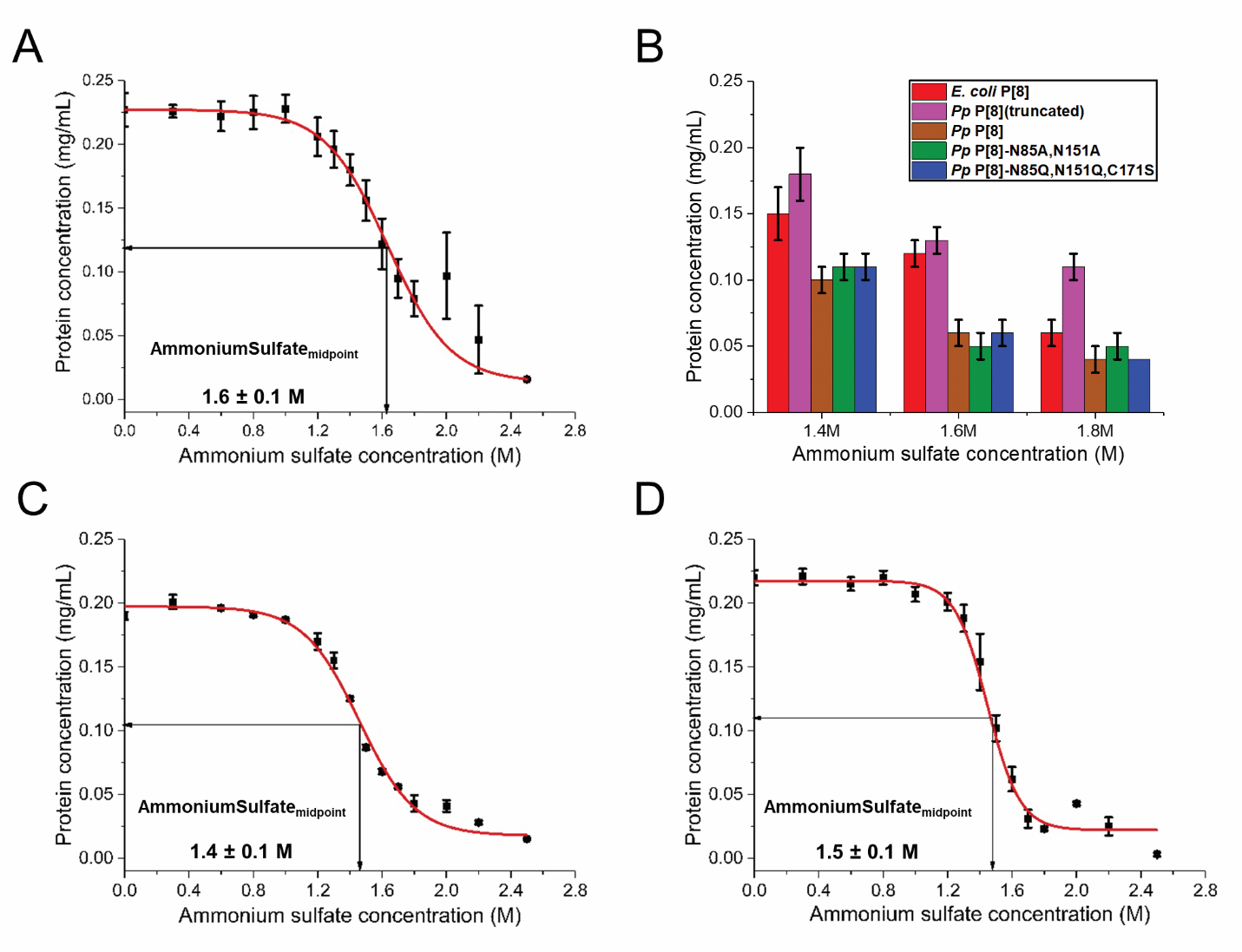
**

**Supplementary Figure S3.** N-terminal truncations of *Pp* P[8] (black) and *Pp* P[8]-N85Q,N151Q,C171S (blue) samples. Intact protein mass analysis of (A) *Pp* P[8] at t=0, t=12 weeks at 4°C (in solution and AH-adsorbed samples); and (B) *Pp* P[8]-N85Q,N151Q,C171S at t=0, t=12 weeks at 4°C (in solution and AH-adsorbed samples).

After 12 weeks of storage at 4°C, in solution *Pp* P[8] samples showed a mixture of full-length *Pp* P[8] species with N-terminal Gln (20,302 Da) and N-terminal Gln converted to pyroGlu (20,285 Da, mass difference of -17 Da) species. The abundance of N-terminal truncation variants (missing varying lengths of P2 region) was also observed to increase over time. For *Pp* P[8]-N85Q,N151Q,C171S in solution samples, a complete loss of full-length species (20,314 Da) concomitant with the appearance of N-terminal truncated variant missing entire flexible P2 region (18,849 Da) was observed. Similar results were observed for AH-adsorbed *Pp* P[8] and *Pp* P[8]-N85Q,N151Q,C171S formulations after 12 weeks of storage, indicating adsorption of NRRV to aluminum did not protect against proteolysis. The average measured masses for all samples were within 0.3-0.4 Da of the theoretical masses. For AH-adsorbed samples, protein was desorbed using 200 mM phosphate before the analysis.


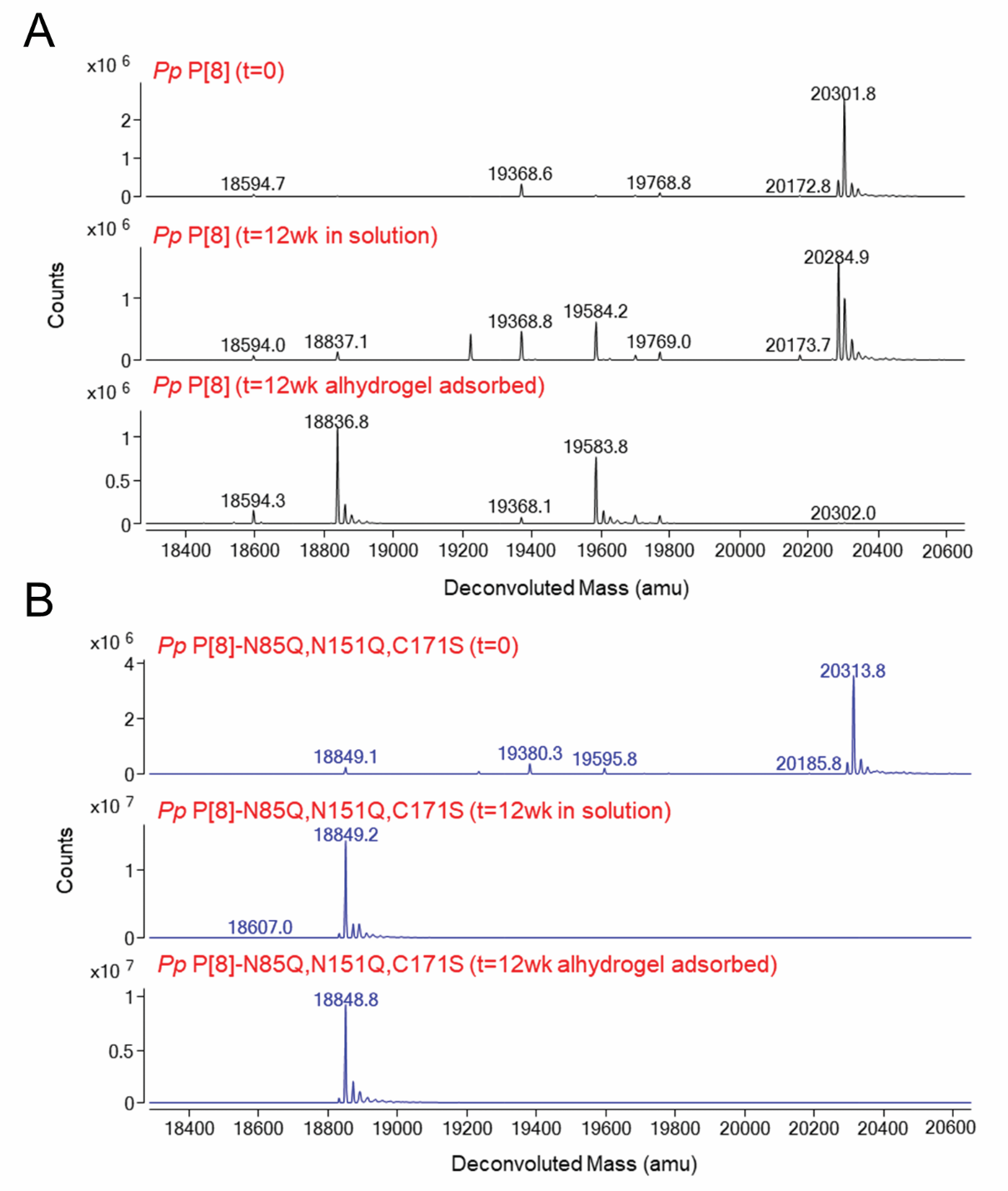


**Supplementary Figure S4.** Effect of N-terminal truncations on antibody binding of *Pp* P[8] (black) and *Pp* P[8]-N85Q,N151Q,C171S (blue) samples. Antibody binding of P[8] stability samples in solution at t=0 and t=12 weeks at 4°C as determined by (A) Octet BLI, and (B) Competitive ELISA; Antibody binding of P[8] samples treated without (-) and with (+) aminopeptidase as determined by (C) Octet BLI, and (D) Competitive ELISA.

Antibody binding analysis of *Pp* P[8] and *Pp* P[8]-N85Q,N151Q,C171S samples by BLI at different time points showed overall similar kinetics with binding affinity ranging within 2-6 nM. Competitive ELISA results indicated ~3-fold increase in antibody binding of *Pp* P[8]-N85Q,N151Q,C171S, while no change was detected for *Pp* P[8] samples over the course of 12 weeks. To determine if absence of P2 region (as observed with *Pp* P[8]-N85Q,N151Q,C171S samples, see Supplementary Figure S3) increases accessibility of the P[8]-specific antibody to the epitope site, we treated *Pp* P[8] and *Pp* P[8]-N85Q,N151Q,C171S samples with aminopeptidase to induce N-terminal cleavage to generate P2-truncated variants. Antibody-binding analysis using BLI showed similar binding affinities of 2-4 nM for all samples, however, ELISA results indicated enhanced antibody-binding by both *Pp* P[8] and *Pp* P[8].


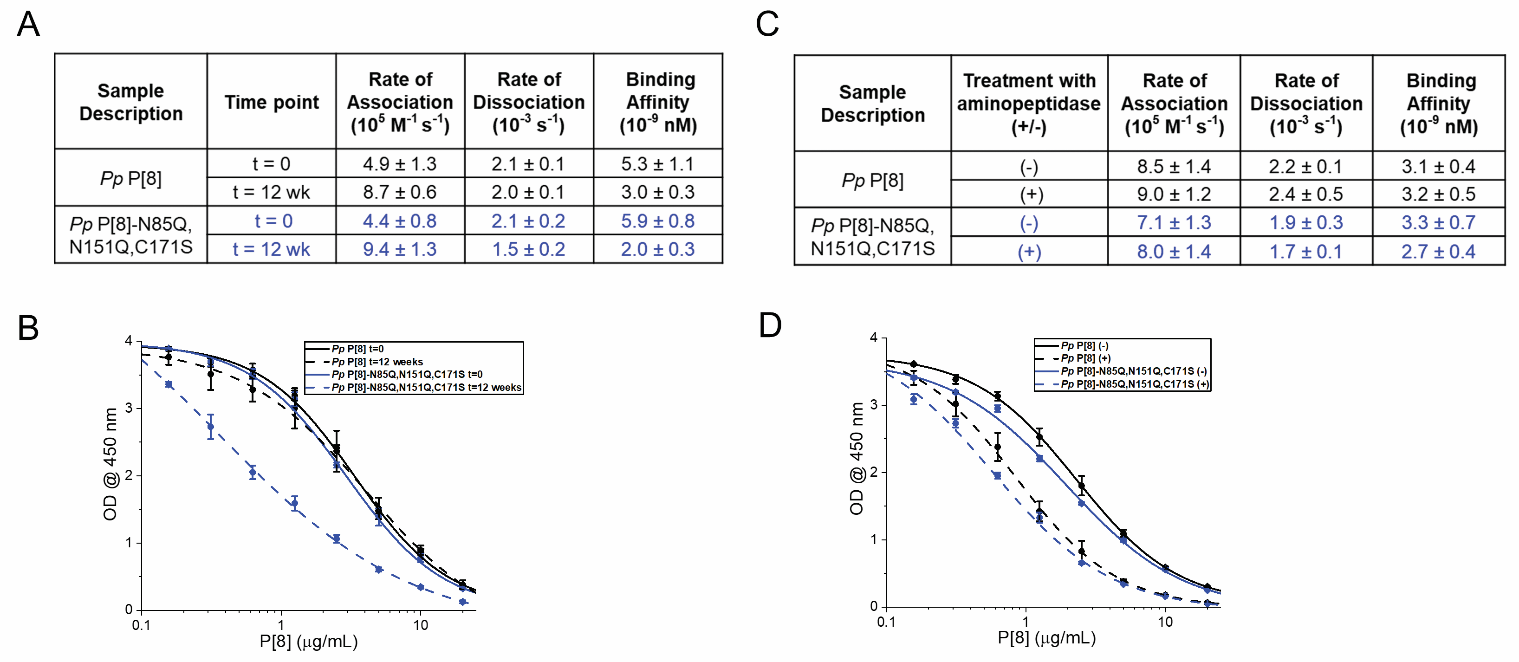

Supplement: Supplemental Information [file mmc1.docx]
